# Failed ventilation via i-gel due to air leakage of the drainage port

**DOI:** 10.1186/s40981-023-00655-3

**Published:** 2023-09-29

**Authors:** Keisuke Kuwana, Makoto Kobayashi, Satoki Inoue

**Affiliations:** Department of Anesthesiology and Division of Intensive Care, 1 Hikarigaoka, Fukushima City, 960-1295 Japan

To the Editor

Supraglottic airway devices (SADs) are commonly used to provide adequate ventilation in anesthesia practice. However, failed ventilation due to incomplete airway management via such devices has been reported, although the incidence is rare [1]. i-gel™ (i-gel, Intersurgical, Berkshire, UK) is a SAD equipped with a gel-like cuff made of thermoplastic elastomer that seals the laryngo-pharyngeal space [2], and has an additional small lumen for esophageal drainage. We herein report a case of failed ventilation because of massive air leakage from an i-gel drainage port. Written informed consent was obtained from the patient for publication of this case report and its accompanying images. A 74-year-old male (height, 163 cm; weight, 69 kg) underwent transurethral resection of bladder tumor under general anesthesia, induction of which was done with remifentanil 0.12 μg/kg/min, fentanyl 100 μg, and propofol 80 mg. Any unique anatomical features had never been pointed out to him. After obtaining muscular relaxation with rocuronium 60 mg, an i-gel #4 was inserted without any specific resistance; however, adequate ventilation was not achieved due to massive air leakage. We attempted to provide manual ventilation with approximately airway pressure of 15 cmH_2_O; however, the target airway pressure was not achieved because of air leakage and each expiratory volume was less than 50 ml. Several attempts were made to properly position the i-gel, but all failed. Close observation revealed that the location of the leakage was a drainage port of the i-gel. Covering the drainage port with a finger significantly reduced the air leakage, and adequate tidal volumes (450–500 ml with airway pressure of 15 cmH_2_O) were achieved. Subsequently, we covered the port with plastic tape, after which adequate airway seal was maintained during surgery.

When ventilating with an i-gel, its distal tip with the distal port of drainage duct is positioned at the esophageal inlet (Figs. 1A and 2A, B). However, in the present case, the distal tip was located at the laryngeal inlet (Figs. 1B and 3A, B). Thus, the inspired air through the ventilation port of the i-gel exited directly through the drainage port located at the laryngeal inlet and did not reach the lungs. An i-gel has a smaller drainage port as well as a smaller distal ventilation port compared to other SADs, which might have allowed the i-gel to migrate into the laryngeal inlet. i-gel might have caused failed ventilation in the current case due to this unique issue. When massive air leakage from the i-gel drainage port is observed, covering the drainage port may be a promising option.

**Fig. 1:**
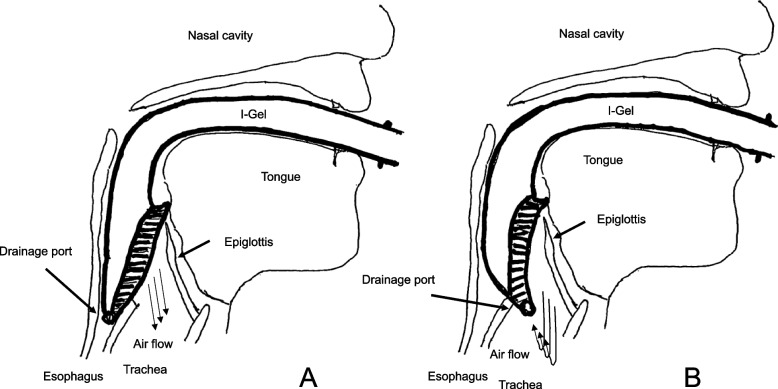
**A**, **B** Illustrations of i-gel placement. **A** Adequate i-gel placement. Ventilated air flows in the direction of the trachea. **B** Inadequate i-gel placement, in which the drainage port at the distal tip is located at the laryngeal inlet. Ventilated air leaks out through the drainage port

**Fig. 2:**
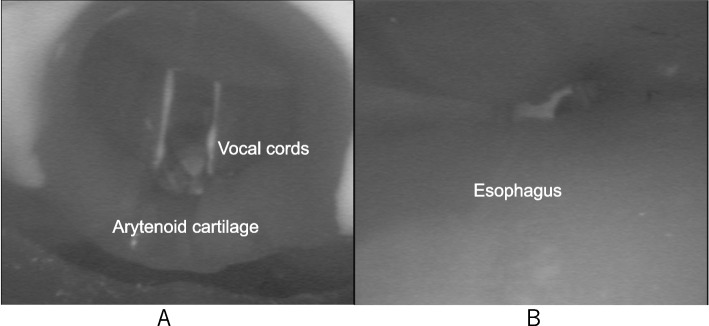
**A**, **B** Bronchoscopic view of deal i-gel placement in a manikin. **A** A view from the distal tip of i-gel ventilation duct. Arytenoid cartilage and vocal cords can be observed. **B** A view from the distal tip of i-gel drainage duct, showing only the esophagus

**Fig. 3:**
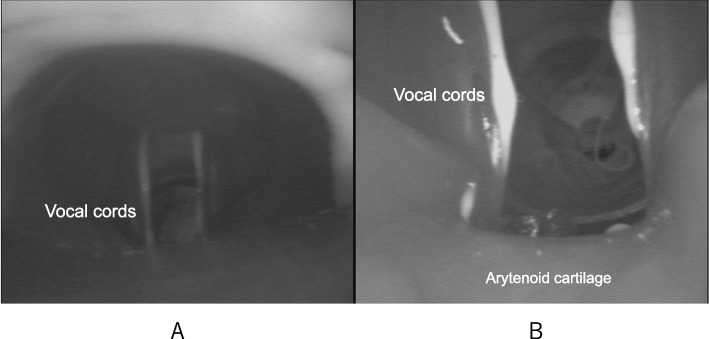
**A**, **B** Bronchoscopic view of approximate i-gel placement in a manikin simulating the present case. **A** A view from the distal tip of i-gel ventilation duct. Vocal cords, but not the arytenoid cartilage, can be observed. **B** A view from the distal tip of i-gel drainage duct. Vocal cords and the edge of the arytenoid cartilage, but not the esophagus, can be observed

## Data Availability

Not applicable.

## Abbreviation

SAD: Supraglottic airway device
